# Trends in cardiovascular risk factors and treatment goals in patients with diabetes in Singapore-analysis of the SingHealth Diabetes Registry

**DOI:** 10.1371/journal.pone.0259157

**Published:** 2021-11-08

**Authors:** Liang Feng, Amanda Lam, David Carmody, Ching Wee Lim, Gilbert Tan, Su-Yen Goh, Yong Mong Bee, Tazeen H. Jafar

**Affiliations:** 1 Program in Health Services & Systems Research, Duke-NUS Medical School, Singapore, Singapore; 2 Department of Endocrinology, Singapore General Hospital, Singapore, Singapore; 3 SingHealth Polyclinics, Singapore, Singapore; 4 Duke Global Health Institute, Durham, NC, United States of America; The Chinese University of Hong Kong, HONG KONG

## Abstract

**Background:**

Asian populations are at high risk of diabetes and related vascular complications. We examined risk factor control, preventive care, and disparities in these trends among adults with diabetes in Singapore.

**Methods:**

The sample included 209,930 adults with diabetes aged≥18 years from a multi-institutional SingHealth Diabetes Registry between 2013 and 2019 in Singapore. We performed logistic generalized estimating equations (GEEs) regression analysis and used linear mixed effect modeling to evaluate the temporal trends.

**Results:**

Between 2013 and 2019, the unadjusted control rates of glycated hemoglobin (4.8%, 95%CI (4.4 to 5.1) and low-density lipoprotein cholesterol (LDL-C) (11.5%, 95%CI (11.1 to 11.8)) improved, but blood pressure (BP) control worsened (systolic BP (SBP)/diastolic BP (DBP) <140/90 mmHg: -6.6%, 95%CI (-7.0 to -6.2)). These trends persisted after accounting for the demographics including age, gender, ethnicity, and housing type. The 10-year adjusted risk for coronary heart disease (CHD) (3.4%, 95% (3.3 to 3.5)) and stroke (10.4%, 95% CI (10.3 to 10.5)) increased. In 2019, the control rates of glycated hemoglobin, BP (SBP/DBP<140/90 mmHg), LDL-C, each, and all three risk factors together, accounted for 51.5%, 67.7%, 72.2%, and 24.4%, respectively.

**Conclusions:**

Trends in risk factor control improved for glycated hemoglobin and LDL-C, but worsened for BP among diabetic adults in Singapore from 2013 to 2019. Control rates for all risk factors remain inadequate.

## Introduction

Diabetes is a major public health problem affecting about 9.3% (463 million) people worldwide. Diabetes increases the risk of macrovascular (e.g. cardiovascular disease (CVD)) and microvascular diseases (e.g. retinopathy and kidney diseases) [1–3], and related mortality [4, 5]. Asian populations are at higher risk of developing type 2 diabetes (T2D), and have a higher risk of complications than their European counterparts [6, 7].

There is substantial evidence that quality improvements at the individual clinical practice, health systems, and policy levels aimed at risk factor control can prevent vascular complications of diabetes [8–10]. It is thus crucial to examine temporal trends in risk factor control to identify areas for improvement in diabetes care, and to inform the development of a more effective health systems response and public health policy. However, information on trends in risk factor control is scarce among individuals with diabetes in Asia.

Therefore, we used data from the SingHealth Diabetes Registry (SDR), a multi-institutional diabetes registry in Singapore, from 2013 to 2019, representing more than 8 healthcare sites and more than 200,000 patients aged ≥18 years with diabetes in Singapore, to examine the temporal trend in risk-factor control and adherence to preventive care. We also sought to evaluate the variation in the trends of risk-factor control across the socio-demographic groups in Singapore.

## Methods

### Population

The multi-institutional SDR was described in detail previously [11]. Briefly, the SDR was established in 2015, and has been populated with data retrospectively and prospectively to cover the period 2013 to 2019. It is updated annually, with patient data from electronic medical records (EMR) across the primary and hospital-based care continuum within SingHealth. The public healthcare system in Singapore is grouped into three major clusters including Singapore Health Services (abbreviated as SingHealth), National Healthcare Group (NHG), and National University Health System (NUHS). Each cluster provides the full suite of healthcare service from primary care to general hospitals and community hospitals. SingHealth, where SDR is based, is the largest cluster and consists of four acute hospitals, five national specialty centers, three community hospitals and eight primary care clinics (SingHealth Polyclinics) [12], which cumulatively provide health services to approximately 50% of the population of Singapore. Patients with diabetes were identified through diagnosis codes (International Classification of Disease, Nine Revision (ICD 9), ICD10, Systematized Nomenclature of Medicine (SNOMED), and SingHealth Polyclinic Working Diagnosis Code), prescription records for diabetes medications, or laboratory tests (fasting plasma glucose, oral glucose tolerance test, or glycated hemoglobin (HbA1c)). The details of each criterion for identifying diabetes have been published earlier [11]. The SDR includes data on patient demographics, prescribed and dispensed medications, co-morbidities, anthropometrics, laboratory tests, and health services utilization. All-cause mortality and CVD mortality data up till December 2019 were obtained by linking the SDR with the Singapore National Death Registry. ICD 10 codes (codes: I00-I99) were used to identify patients for whom CVD was the primary diagnosis and listed as underlying cause of death. The present study included patients with both type 1 and type 2 diabetes aged ≥ 18 years, enrolled between 2013 and 2019 (n = 209,930). More than 95% had type 2 diabetes. The study was approved by the NUS Institutional Review Board and SingHealth Centralised Institutional Review Board. Informed consent was not sought from patients because the study analyzed anonymised datasets from the SDR in a sandbox environment.

### Measurement

Data extracted from SDR were socio-demographics, clinical profiles, and laboratory tests. For each patient, annual average was calculated for continuous clinical data with multiple measurements (e.g. blood pressure (BP)) in a year. Sociodemographic data included age (stratified into 18–44 years, 45–64 years, ≥65years), gender, ethnicity (Chinese, Indian, Malay, and others), and housing type (1 to 2- room Housing Development Board (HDB) flat, 3 to 5-room HDB flat, condominium or landed property). HDB flats are subsidised public housing for local residents.

Data on clinical profiles included body mass index (BMI), systolic blood pressure (SBP), diastolic blood pressure (DBP), medication use, duration and complications of diabetes, and laboratory measurements. BMI was dichotomized using 23 kg/m^2^ as the cut point. Target BP was defined as SBP/DBP≤140/90 mmHg and ≤130/80 mmHg. Duration of diabetes was grouped into <5 years, 5 to 15 years, and ≥15 years. Complications including both macro-and microvascular complications were dichotomized as with or without complications in the study.

Laboratory test data included HbA1c, total cholesterol (TC), high-density lipoprotein cholesterol (HDL-C), low-density lipoprotein cholesterol (LDL-C), and urine albumin to creatinine ratio (UACR). We defined poorly-controlled, moderately-controlled, and well-controlled glycemia as HbA1c level of >9.0%, <8.0%, and <7.0% respectively based on recommended standards [13–15]. The target for LDL-C was <100 mg/dL among all patients and <70 mg/dL among those with pre-existing CVD. We also assessed the proportion that simultaneously attained goals (combined target) for HbA1c (<7.0%), BP (< 140/90 mmHg or < 130/80mmHg) and LDL-C (<100 mg/dl). Based on previous reports [16–18], we also defined individualized HbA1c targets according to risk profile: patients aged <45 years without complications (HbA1c ≤6.5%) or with complications (HbA1c ≤7.0%), patients aged 45 to 64 years without complications (HbA1c ≤7.0%) or with complications (HbA1c ≤8.0%), and patients aged ≥65 years without complications (HbA1c ≤7.0%) or with complications (HbA1c ≤8.0%). To evaluate benefits of optimizing diabetes care, proportion of patients without microalbuminuria (UACR<30 mg/g) and 10-year risk of coronary heart disease (CHD) and stroke were calculated using the UK Prospective Diabetes Study (UKPDS) risk engine [19, 20]. We defined a high 10-year risk of CHD or stroke as a risk score of ≥15%.

The risk factors assessed were HbA1c, BP, lipids (LDL-C, HDL-C, and TC), and BMI using targets as defined above. Patient adherence to preventive care was assessed by annual measurement of LDL-C and UACR, and receipt of renin angiotensin aldosterone system (RAAS) inhibitors, an angiotensin-converting–enzyme (ACE) inhibitor or angiotensin-receptor blocker (ARB), when UACR ≥30 mg/g. Medication use included the prescriptions of glucose-lowering medications (metformin, Sodium-glucose co-transporter-2 inhibitor (SGLT2 i), and insulin), antihypertensive medication, statin, and antiplatelet therapy.

### Statistical analysis

Categorical and continuous variables were reported for each year from 2013 to 2019 in number and percentage, and mean and standard deviation, respectively.

Both univariate and multivariate logistic and linear regression analysis were done to assess the temporal trend of risk factor control, preventive care, and medication use, depending on the nature of the outcomes. Generalized estimating equations (GEEs) were used for binary outcomes with an unstructured working correlation matrix to account for within-patient correlations. For continuous outcomes, linear mixed effect models were used to include a random intercept to account for within-patient correlations. In both univariate and multivariate analyses, year of data collection was modeled as a categorical variable to compute the differences and their 95% CIs in the proportions or mean of risk factor control, preventive care, and medication use between 2013 and 2019. The covariates controlled for in the multivariate model were age, gender, ethnicity, and housing type. Analysis for each outcome of meeting the control target of HbA1c (<7.0%), BP (SBP/DBP<140/90 mmHg), LDL-C (<100 mg/dl), and all the three risk factors combined was additionally adjusted for use of antidiabetic medication, use of antihypertensives, use of lipid-lowering medication, and all the three variables of medication use, respectively, and was then adjusted for BMI.

Linear trend was evaluated by modelling year of data collection as a continuous variable. Analysis was also done for the outcome of medication use including the use of glucose-lowering medications (metformin, SGLT2 i, and insulin), antihypertensive medications, and statin among patients with diabetes having uncontrolled risk factors.

We also used logistic GEEs regression with an unstructured working correlation matrix to examine whether sociodemographic profiles might alter any observed changes in risk factor control and 10-year CHD or stroke risk between 2013 and 2019. In the regression model, control of different risk factors (controlled vs. uncontrolled) and 10-year CHD or stroke risk (high vs. low) were the dependent variables, and the covariates were year of data collection, age, gender, ethnicity, and housing type. The interactions were evaluated separately between year of data collection and the other covariates (i.e. age, gender, ethnicity, and housing type) for each outcome. Moreover, the analysis was repeated for the outcomes of meeting control target of different risk factors (i.e. individualized HbA1c, BP, HDL-C, combination of the three) by further controlling for medication use and BMI in the models.

Descriptive results and analysis of linear mixed effect models were done using R software version 3.6.0 via RStudio Server version 1.3. 959. Logistic GEEs regressions were done using STATA version 13.0 (Stata Corporation, College Station, TX, USA). A two-sided p value of <0.05 was considered statistically significant.

## Results

### Socio-demographics of patients with diabetes

Data from 209,930 unique patients were available in the SDR between 2013 and 2019. We excluded patients with illogical data (n = 9) and if data for all of the following three measurements were missing: BP, HbA1c, and LDL-C (n = 15,119). The final analytic dataset included 194,802 patients with 756,864 records for the final analysis.

The distribution of gender, ethnicity, and housing type remained stable between 2013 and 2019, but the proportions of the elderly (≥65 years) (51.7% in 2013 vs 56.2% in 2019, p<0.001) and those with a long duration of diabetes (≥12 years) (30.2% in 2013 vs 42.4% in 2019, p<0.001) increased markedly during this period. (Table 1)

**Table 1:** Characteristics of patients with diabetes.

Characteristics	2013	2014	2015	2016	2017	2018	2019
	(n = 86480)	(n = 93132)	(n = 99404)	(n = 106144)	(n = 110623)	(n = 127901)	(n = 133180)
Age(yr), n(%)							
18–44	4181 (4.8)	4601 (4.9)	4916 (4.9)	5306 (5.0)	5016 (4.5)	7434 (5.8)	7728 (5.8)
45–64	37612 (43.5)	39401 (42.3)	41210 (41.4)	43401 (40.9)	42441 (38.4)	49974 (39.1)	50552 (38.0)
≥65	44687 (51.7)	49130 (52.8)	53278 (53.7)	57437 (54.1)	63166 (57.1)	70493 (55.1)	74900 (56.2)
Female, n(%)	43305 (50.1)	46450 (49.9)	49238 (49.5)	52325 (49.3)	54821 (49.6)	62590 (48.9)	64908 (48.7)
Ethnicity, n(%)							
Chinese	61375 (71.0)	65968 (70.8)	70341 (70.8)	74892 (70.6)	78494 (71.0)	89929 (70.3)	93685 (70.3)
Indian	9014 (10.4)	9673 (10.4)	10310 (10.4)	11089 (10.4)	11613 (10.5)	13307 (10.4)	13715 (10.3)
Malay	12467 (14.4)	13616 (14.6)	14584 (14.7)	15686 (14.8)	15903 (14.4)	19078 (14.9)	19861 (14.9)
Others	3624 (4.2)	3875 (4.2)	4169 (4.2)	4477 (4.2)	4613 (4.2)	5587 (4.4)	5919 (4.4)
Housing type, n(%)							
1~2 rooms HDB	7046 (8.5)	7455 (8.4)	7830 (8.3)	8189 (8.1)	8296 (7.9)	9259 (7.7)	9325 (7.5)
3~5 rooms HDB	66814 (80.7)	71742 (80.6)	76428 (80.6)	81269 (80.5)	84441 (80.3)	96792 (80.5)	100375 (80.6)
Condo or landed house	8901 (10.8)	9791 (11.0)	10569 (11.1)	11525 (11.4)	12448 (11.8)	14219 (11.8)	14897 (12.0)
Missing	3538	4049	4500	5096	5387	7576	8583
Time since diabetes diagnosis (yr), (%)							
0 to <5	17467 (32.0)	18157 (30.7)	19114 (30.0)	20792 (30.4)	18910 (27.4)	19789 (26.8)	15293 (21.4)
5 to <12	20640 (37.8)	22230 (37.6)	23399 (36.8)	24211 (35.4)	24102 (34.9)	25567 (34.6)	25938 (36.2)
≥12	16537 (30.2)	18794 (31.8)	21081 (33.2)	23283 (34.1)	26052 (37.7)	28580 (38.6)	30385 (42.4)
Missing	31836	33951	35810	37858	41559	53965	61564

Abbreviation: HDB, Housing and Development Board

### Changes in risk factor control

From 2013 to 2019 (Table 2), the unadjusted mean HbA1c, DBP, LDL-C, HDL-C, TC, and BMI decreased by 0.2% (95%CI (0.2,0.2)), 0.9 mmHg (95%CI (0.9, 1.0)), 11.7 mg/dl (95%CI(11.5,11.9)), -1.3 mg/dl (95%CI(-1.4, -1.2)), -14.5 mg/dl (95%CI (-14.8, -14.2)), 0.6 kg/m^2^ (95%CI(0.5, 0.6)), respectively. However,the unadjusted mean SBP increased by 2.4 (95%CI (2.3, 2.5)) mmHg.

**Table 2:** Change in risk factor control and preventive care among patients with diabetes.

Characteristics	2013	2014	2015	2016	2017	2018	2019	Unadjusted Change from 2013 to 2019, mean or % (95% CI)^a^	Adjusted Change from 2013 to 2019, mean or % (95% CI)^b^	P for linear trend^c^
	(n = 86480)	(n = 93132)	(n = 99404)	(n = 106144)	(n = 110623)	(n = 127901)	(n = 133180)			
**HbA1c**										
Mean (SD)	7.4(1.5)	7.3 (1.4)	7.4 (1.4)	7.4 (1.4)	7.5 (1.4)	7.2 (1.4)	7.2 (1.4)	-0.2 (-0.2, -0.2)	-0.1 (-0.1, -0.1)	<0.001
>9.0%, n/N (%)	9428/81535 (11.6)	9208/84971 (10.8)	10072/90129 (11.2)	10155/95026 (10.7)	10386/95239 (10.9)	11120/115123 (9.7)	11308/120947 (9.3)	-2.7 (-2.9 to -2.5)	-1.9 (-2.1, -1.7)	<0.001
<8.0%, n/N (%)	62367/81535 (76.5)	65888/84971 (77.5)	68777/90129 (76.3)	72711 /95026 (76.5)	71607/95239 (75.2)	91640/115123 (79.6)	96417/120947 (79.7)	3.5 (3.2 to 3.8)	2.3 (2.0, 2.6)	<0.001
<7.0%, n/N (%)	37407/81535 (45.9)	41239/84971 (48.5)	41977/90129 (46.6)	42906/95026 (45.2)	37424/95239 (39.3)	59649/115123 (51.8)	62237/120947 (51.5)	4.8 (4.4 to 5.1)	3.5 (3.1, 3.9)	<0.001
Missing, n	4945	8161	9275	11118	15384	12778	12233			
**Blood pressure (mmHg)**										
SBP, mean (SD)	132 (14.3)	133 (14.7)	133 (14.6)	133 (14.1)	134 (14.4)	135 (14.4)	135 (14.7)	2.4 (2.3, 2.5)	2.1 (2.0, 2.2)	<0.001
DBP, mean (SD)	70.1 (8.4)	70.1 (8.4)	70.2 (8.1)	70.0 (8.0)	70.0 (7.8)	70.8 (8.0)	71.2 (8.2)	-0.9 (-1.0, -0.9)	-0.3 (-0.4,-0.3)	<0.001
<130/80, n/N (%)	42356/77465 (45.3)	49995/ 87119 (42.6)	37540/ 92378 (40.6)	40126/ 101166 (39.7)	41216/ 109027 (37.8)	45451/ 126254 (36.0)	47515/130968 (36.3)	-7.5 (-7.8 to -7.1)	-6.9 (-7.3, -6.6)	<0.001
<140/90, n/N (%)	58092/77465 (75.0)	63502/87119 (72.9)	66350/92378 (71.8)	72988/101166 (72.1)	76536/ 109027 (70.2)	86562/126254 (68.6)	88653/130968 (67.7)	-6.6 (-7.0 to -6.2)	-6.0 (-6.4,-5.6)	<0.001
Missing, n	9015	6013	7026	4978	1596	1647	2212			
**LDL cholesterol (**mg/dl)										
Mean (SD)	92.9 (28.9)	94.2 (29.4)	93.0 (29.1)	90.2 (28.4)	84.6 (27.7)	85.9 (29.2)	86.1 (29.4)	-11.7 (-11.9, -11.5)	-10.2 (-10.4,-10.1)	<0.001
<100, n/N (%)	48008/74074 (64.8)	47918/ 76064 (63.0)	52562/ 81748 (64.3)	59039/ 86870 (68.0)	66566/ 88821 (74.9)	75516/ 103908 (72.7)	78056/108072 (72.2)	11.5 (11.1 to 11.8)	10.1 (9.7, 10.5)	<0.001
Missing, n	12406	17068	17656	19274	21802	23993	25108			
**LDL cholesterol (mg/dl) among patients with CVD** ^d^										
Mean (SD)	90.4 (29.3)	91.1(30.0)	89.5 (29.8)	85.1 (28.7)	81.2 (28.5)	81.6(30.0)	80.4 (30.2)	-12.0 (-12.4, -11.5)	-11.4 (-11.8, -11.0)	<0.001
<70, n/N (%)	5646/21126 (26.7)	5885/22515 (26.1)	6905/ 24543 (28.1)	9302/ 27390 (34.0)	11931/29333 (40.7)	14581/ 35100 (41.5)	16073/37047 (43.4)	19.4 (18.6 to 20.1)	18.4 (17.6,19.2)	<0.001
Missing, n	3905	4957	5518	6381	8021	7823	8345			
**HbA1c, blood pressure, and LDL cholesterol**										
Targets achieved ^e^, n/N (%)	11128/80904 (13.8)	11403/ 86968 (13.1)	11139/ 92932 (12.0)	11604/ 99703 (11.6)	10889/ 104068 (10.5)	15444/ 120808 (12.8)	15864/125653 (12.6)	-0.4 (-0.6 to -0.1)	-0.7 (-0.9, -0.4)	<0.001
Missing ^e^, n	5576	6164	6472	6441	6555	7093	7527			
Targets achieved ^f^ n/N (%)	17733/79231 (22.4)	18793/ 84035 (22.4)	19115/89880 (21.3)	20521/ 96075 (21.4)	19565/99611 (19.6)	29625/ 116328 (25.5)	29534/121152 (24.4)	3.0 (2.7 to 3.4)	2.2 (1.8, 2.5)	<0.001
Missing ^f^, n	7249	9097	9524	10069	11012	11573	12028			
HDL cholesterol (mg/dl)										
Mean (SD)	50.8 (13.8)	50.7 (13.9)	50.1 (13.7)	50.2 (13.9)	50.4 (14.1)	50.3 (14.0)	49.4 (13.1)	-1.3 (-1.4, -1.2)	-1.5 (-1.6, -1.4)	<0.001
Missing, n	11797	16463	17013	18696	21424	23454	24425			
Total cholesterol (mg/dl)										
Mean (SD)	171.0 (35.1)	172.5 (35.7)	170.8 (35.2)	167.9 (34.6)	161.5 (33.4)	162.3 (35.0)	161.3 (35.3)	-14.5 (-14.8, -14.2)	-13.0 (-13.3, -12.7)	<0.001
Missing, n	11782	16428	16996	18633	21322	23334	24398			
**BMI (kg/m** ^**2**^ **)**										
Mean (SD)	26.3 (4.6)	26.5 (4.6)	26.6 (4.8)	26.4 (4.7)	26.3 (4.7)	26.4 (4.9)	26.4 (4.9)	-0.6 (-0.6, -0.5)	-0.5 (-0.6, -0.5)	<0.001
<23, **n**/N (%)	7172/31510 (22.8)	16201/71946 (22.5)	8081/36472 (22.2)	21358/90493 (23.6)	24857/101053 (24.6)	27944/116270 (24.0)	29842/121377 (24.6)	5.0 (4.7, 5.4)	3.9 (3.5, 4.2)	<0.001
Missing, n	54970	21186	62932	15651	9570	11631	11803			
**Annual LDL measurement,** n (%)	74074 (85.7)	76064 (81.7)	81748 (82.2)	86870 (81.8)	88821 (80.3)	103908 (81.2)	108072 (81.1)	-6.7 (-7.1 to -6.3)	-6.2 (-6.6, -5.8)	<0.001
**ACEI or ARB, if UACR ≥30 mg/g**^g^, n/N (%)	1998/ 2460 (81.2)	1932/2341 (82.5)	1820/2278 (79.9)	2134/2666 (80.0)	12960/16017 (80.9)	27279/34566 (78.9)	26090/31518 (82.8)	2.0 (0.5 to 3.6)	0.2 (-1.4, 1.9)	<0.001
**UACR**										
Available UACR measurement, n (%)	4870 (5.6)	4718 (5.1)	4597 (4.6)	5227 (5.0)	37363 (33.8)	82990 (64.9)	67652 (50.8)	41.5 (41.2 to 41.9)	42.0 (41.6, 42.3)	<0.001
<30 mg/g, n/N (%)	2410/ 4870 (49.5)	2377/4718 (50.4)	2319/4579 (50.4)	2561/5227 (49.0)	21346/ 37363 (57.1)	48424/ 82990 (58.3)	36134/67652 (53.4)	-0.4 (-1.5 to 0.8)	1.9 (0.7, 3.2)	<0.001
Missing, n	81610	88414	94807	100917	73260	44911	65528			
Atrial fibrillation, n/N (%)	2966/86471 (3.4)	4014/91812 (4.4)	4752/98076 (4.8)	5640/105128 (5.4)	6651/109734 (6.1)	8093/127026 (6.4)	9000/133180 (6.8)	8.8 (8.4, 9.1)	7.8 (7.5, 8.2)	<0.001
Missing, n	9	1320	1328	1016	889	875	0			
**10-yr UKPDS CHD risk**										
Mean (SD)	15.5 (13.3)	16.0 (13.7)	16.6 (13.9)	16.3 (13.7)	16.5 (14.1)	15.7 (13.7)	16.3 (13.8)	4.4 (4.3, 4.5)	3.4 (3.3, 3.5)	<0.001
Missing, n	48632	52671	55657	58248	61903	75115	83862			
**10-yr UKPDS stroke risk**										
Mean (SD)	11.9 (14.4)	12.9 (15.9)	13.8 (16.9)	14.1 (17.5)	15.1 (18.5)	15.4 (18.8)	16.8 (19.9)	10.9 (10.8, 10.9)	10.4 (10.3, 10.5)	<0.001
Missing, n	44220	47612	49818	51630	54616	67141	75692			
**CVD mortality**, n/N (%)	729/86480 (0.84)	1042/93849 (1.11)	1119/10029 (1.12)	1295/107290 (1.21)	1391/111789 (1.24)	1625 /129263 (1.26)	1944 /134937 (1.44)	0.80 (0.65,0.95)	0.71 (0.56,0.85)	<0.001
**All-cause mortality, n/N (%)**	1905/86480 (2.2)	2773/93849 (2.9)	3164/100299 (3.2)	3668/107290 (3.4)	4016/111789 (3.6)	5325/129263 (4.1)	6210/134937 (4.6)	3.5 (3.2, 3.8)	3.1 (2.9, 3.4)	<0.001

N was the total number of patients with the corresponding measurement

Abbreviation: 95% CI, 95% confidence interval; SD, standard deviation; SBP, systolic blood pressure; DBP, diastolic blood pressure; LDL, low density lipoprotein; HDL, high density lipoprotein; CVD, cardiovascular disease; BMI, body mass index; ACEI, Angiotensin-converting enzyme inhibitor; ARB, angiotensin-receptor blocker; UACR, urine albumin and creatinine ratio; UKPDS, United Kingdom Prospective Diabetes Study; CHD, coronary heart disease

a Predictive margins were calculated from univariate linear mixed effect models or logistic generalized estimating equations (GEEs) regression with categorical year of data collection as the independent variable

b Predictive margins were calculated using multivariate linear mixed effect models or logistic generalized estimating equations (GEEs) regression, including categorical year of data collection and adjusting for age, gender, ethnicity, and housing type.

c P value for linear trend was calculated using multivariate linear mixed effect model or logistic generalized estimating equations (GEEs) with continuous year of data collection, age, gender, ethnicity, and housing type as the independent variables

d Analysis limited to patients with CVD

e HbA1c<7.0%, SBP≤130&DBP≤80 mmHg, and LDL<100mg/dl

f HbA1c<7.0%, SBP≤140&DBP≤90 mmHg, and LDL<100mg/dl

g Analysis limited to patients with ACR ≥30 mg/g

**Fig 1:**
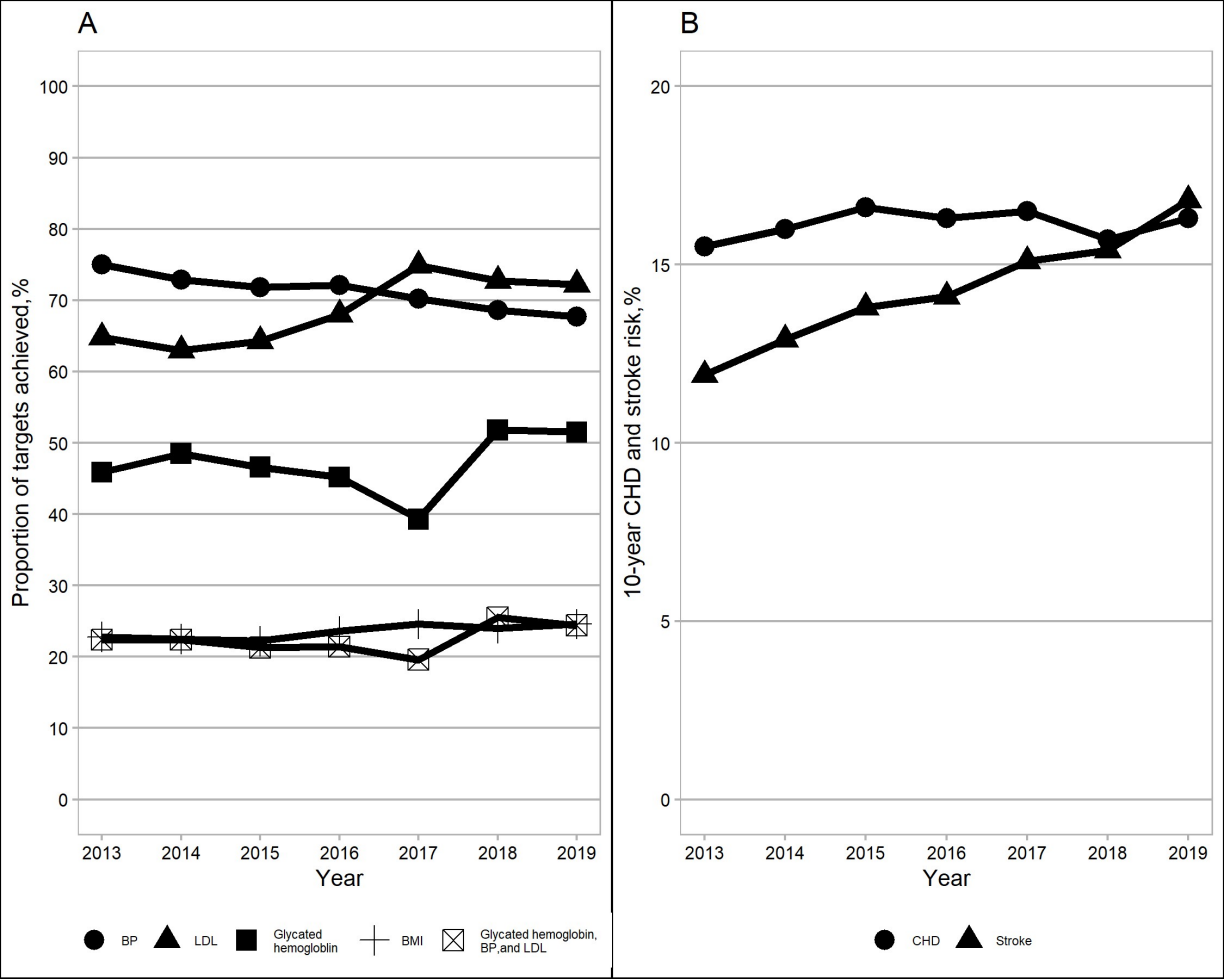
Risk-factor control and 10-year risk for coronary heart disease (CHD) and stroke among patients with diabetes from 2013 to 2019. (A) risk factors included HbA1c (<7.0%), blood pressure (BP) (systolic /diastolic BP<140/90 mmHg), low-density lipoprotein cholesterol (LDL-C) (<100 mg/dl), body mass index (BMI) (<23 kg/m^2^), as well as the combination of HbA1c, BP, and LDL-C. (B) 10-year risk for CHD and stroke.

During the same period (Table 2 and Fig 1A), a significant increase was observed in the unadjusted proportion of patients achieving HbA1c <7.0% (4.8%, 95%CI (4.4 to 5.1), P for linear trend <0.001) and LDL-C <100mg/dl (11.5%, 95%CI (11.1 to 11.8), P for linear trend <0.001 among the entire cohort patients; 19.4%, 95%CI (18.6 to 20.1), P for linear trend <0.001 among those with CVD), and in the proportion of BMI<23 kg/m^2^ (5.0%, 95%CI (4.7 to 5.4), P for linear trend <0.001). However, the unadjusted BP control rate decreased significantly from 2013 to 2019 (SBP/DBP<130/80 mmHg: -7.5%, 95%CI (-7.8 to -7.1), P for linear trend <0.001; SBP/DBP<140/90 mmHg: -6.6%, 95%CI (-7.0 to -6.2), P for linear trend <0.001). These temporal trends in risk factor control did not change after adjustment for age, gender, ethnicity, housing type, medication use, and BMI (Tables 2 and S1). In 2019, the proportion of patients meeting individual HbA1c, BP, LDL-C, and combined targets accounted for 51.5%, 67.7%, 72.2%, and 24.4%, respectively.

### Changes in albuminuria, atrial fibrillation, CVD risk, and mortality

Only 5.6% of the cohort had available UACR data in 2013 as the semi-quantitative dipstick test for urine microalbumin was the prevalent test used in the primary care clinics then. With the availability of UACR test in primary care clinics, this proportion increased by eight times to 50.8% in 2019 (Table 2). A slight increase was observed in the proportion of people with UACR <30 mg/g. Atrial fibrillation increased by 7.8% (95%CI (7.5 to 8.2), P for linear trend <0.001) from 2013 to 2019. Both the 10-year CHD risk (adjusted change:3.4%, 95% CI (3.3 to 3.5), P for linear trend <0.001) and 10-year stroke risk (adjusted change:10.4%, 95% CI (10.3 to 10.5), P for linear trend <0.001) increased in 2019 as compared with 2013 (Table 2 and Fig 1B). Also, a gradual increase between 2013 and 2019 was observed in both all-cause (adjusted change:3.1%, 95%CI (2.9, 3.4), P for linear trend <0.001) and CVD mortality (adjusted change:0.71%, 95% CI (0.56, 0.85), P for linear trend <0.001).

### Changes in preventive care

In 2019, relative to 2013, the adjusted proportion of annual LDL-C checks decreased by 6.2% (95%CI (5.8 to 6.6), P for linear trend <0.001), and the adjusted use of ACEI or ARB increased by 0.2% (95%CI(-1.4 to 1.9), P for linear trend <0.001) among individuals with UACR ≥ 30mg/g (Table 2).

### Changes according to risk group

When individualized, risk-specific targets were used for HbA1c (Table 3), greater improvement in glycemic control was observed in the subgroups aged ≤65 years, and the control rate stayed constant over time among the individuals older than 65 years with complications. In the entire cohort, the adjusted individualized glycemic control rate increased by 6.0% (95%CI, (5.6 to 6.3), P for linear trend <0.001), and about 69% were at the individualized target for HbA1c in 2019.

**Table 3:** Diabetic adults with HbA1c levels meeting individualized targets, according to age and presence or absence of complications.

		2013		2014		2015		2016		2017		2018		2019		Unadjusted change from 2013 to 2019,	Adjusted change from 2013 to 2019,	P for linear trend^c^
																% (95% CI) ^a^	% (95% CI) ^b^	
Age and complication Status	Target glycated haemoglobin level, %	N of population	Target met	N of population	Target met	N of population	Target met	N of population	Target met	N of population	Target met	N of population	Target met	N of population	Target met			
			n %		n %		n %		n %		n %		n %		n %			
18–44 yr without complications	≤6.5	3010	573 (19.0)	3030	659 (21.7)	3182	673 (21.1)	3310	732 (22.1)	2663	555 (20.8)	3891	1360 (35.0)	4978	1631 (32.8)	13.7 (11.6 to 15.9)	11.9 (9.6, 14.1)	<0.001
18–44 yr with complications	≤7.0	887	211 (23.8)	915	222 (24.3)	1059	258 (24.4)	1166	296 (25.4)	1335	341 (25.5)	2247	849 (37.8)	1560	587 (37.6)	16.0 (12.0 to 20.0)	16.5 (12.3, 20.8)	<0.001
45–64 yr without complications	≤7.0	22725	9953 (43.8)	22827	10812 (47.4)	23354	10619 (45.5)	23534	10412 (44.2)	19412	7681 (39.6)	21063	11806 (56.1)	26512	14186 (53.5)	7.7 (6.9 to 8.5)	7.6 (6.8, 8.4)	<0.001
45–64 yr with complications	≤8.0	12718	8273 (65.0)	13173	8729 (66.3)	14177	9181 (64.8)	15555	10164 (65.3)	17841	11604 (65.0)	24468	17424 (71.2)	20058	14323 (71.4)	7.9 (7.1 to 8.7)	7.8 (7.0, 8.6)	<0.001
≥65 yr without complications	≤7.0	17486	10727 (61.3)	18565	12048 (64.9)	19548	12288 (62.9)	19856	12263 (61.8)	17821	9620 (54.0)	17554	12120 (69.0)	23895	15286 (66.2)	3.3 (2.5 to 4.1)	3.2 (2.4, 4.0)	<0.001
≥65 yr with complications	≤8.0	24709	20702 (83.8)	26461	22175 (83.8)	28809	23702 (82.3)	31605	25997 (82.3)	36167	28999 (80.2)	45899	38578 (84.0)	43944	36741 (83.6)	0.1 (-0.5 to 0.6)	0.0 (-0.5, 0.5)	0.845
All adults ≥18 yr	Individualized ^d^	81535	50439 (61.9)	84971	54645 (64.3)	90129	56721 (62.9)	95026	59864 (63.0)	95239	58800 (61.7)	115122	82137 (71.3)	120947	83294 (68.9)	8.0 (7.6 to 8.3)	6.0 (5.6, 6.3)	<0.001

Abbreviation: 95% CI, 95% confidence interval

a Predictive margins were calculated from univariate logistic generalized estimating equations (GEEs) regression for correlated outcomes with categorical year of data collection as the independent variable

b Predictive margins were calculated from multivariate logistic generalized estimating equations (GEEs) regression for correlated outcomes with categorical year of data collection, age (only for all adults≥18 yr), gender, ethnicity, and housing type as the independent variables

c P value for linear trend was calculated using multivariate logistic generalized estimating equations (GEEs) regression for correlated outcomes with continuous year of data collection, age (only for all adults≥18 yr), gender, ethnicity, and housing type as the independent variables

d Participants younger than 45 years of age without complications (HbA1c level, ≤6.5%) or with complications (≤7.0%); those 45 to 64 years of age without complications (HbA1c level, ≤7.0%) or with complications (≤8.0%); and those 65 years of age or older without complications (HbA1c level, ≤7.0%) or with complications(≤8.0%).

Tables 4 and S2 show adjusted temporal trends in risk factor control, 10-year CHD and stroke risk, CVD mortality by various subgroups of demographics based on the logistic GEEs regression model. Compared with younger individuals, a greater proportion of older adults aged ≥ 65 years achieved their individualized HbA1c and LDL-C targets, and combined target in 2013. However, a smaller proportion of adults aged ≥ 65 years achieved BP targets in 2013, and older adults also had higher 10-year CHD and stroke risks in 2013 compared to younger individuals. The subgroup of adults aged ≥ 65 years also experienced smaller improvements in the proportions achieving HbA1c, LDL-C and combined targets over time except for BP, and had comparatively greater increases in 10-year CHD and stroke risks over time.

**Table 4:** Adjusted change between 2013 and 2019 in proportions a (95% confidence interval) achieving single or combined risk factor control among patients with diabetes according to demographic characteristics.

Characteristics	Met individualized HbA_1c_ target ^b^ (n = 160178)				BP<140/90 mmHg				LDL-C<100mg/dL				Controlled all 3 risk factors^c^				BMI<23kg/m2			
					(n = 176809)				(n = 150692)				(n = 164305)				(n = 161279)			
	Year 2013, %	Year 2019, %	Absolute change from 2013 to 2019, % (95% CI)	P for interaction ^d^	Year 2013,%	Year 2019, %	Absolute change from 2013 to 2019, % (95% CI)	P for interaction ^d^	Year 2013, %	Year 2019, %	Absolute change from 2013 to 2019, % (95% CI)	P for interaction ^d^	Year 2013, %	Year 2019, %	Absolute change from 2013 to 2019, % (95% CI)	P for interaction ^d^	Year 2013, %	Year 2019, %	Absolute change from 2013 to 2019, % (95% CI)	P for interaction ^d^
Age (yr)				<0.001				<0.001				0.001				<0.001				<0.001
18~44	28.6	36.3	7.7 (6.4 to 8.9)		80.7	77.5	-3.2 (-4.3 to -2.1)		37.9	50.0	12.1 (10.6 to 13.5)		7.3	9.9	2.6 (1.8 to 3.4)		13.0	13.4	0.4 (-0.3 to 1.1)	
45~64	56.0	63.5	7.5 (7.0 to 8.0)		75.2	70.8	-4.4 (-4.9 to -3.9)		54.3	66.1	11.8 (11.3 to 12.3)		24.1	30.0	5.9 (5.4 to 6.4)		19.7	22.2	2.5 (2.1 to 2.9)	
65 and over	71.6	76.4	4.8 (4.4 to 5.2)		69.9	62.5	-7.4 (-7.8 to -6.9)		67.1	75.8	8.7 (8.4 to 9.1)		32.4	34.7	2.3 (1.8 to 2.8)		25.7	30.7	5.0 (4.6 to 5.4)	
Gender				0.042				<0.001				<0.001				<0.001				0.48
Male	63.8	69.9	6.1 (5.7 to 6.6)		73.7	68.3	-5.4 (-5.9 to -4.9)		62.2	72.5	10.3 (9.9 to 10.7)		29.7	34.2	4.5 (4.0 to 5.0)		22.2	26.1	3.9 (3.5 to 4.3)	
Female	62.7	68.5	5.8 (5.4 to 6.2)		71.4	64.8	-6.6 (-7.1 to -6.1)		59.1	69.0	9.9 (9.4 to 10.3)		26.1	29.0	2.9 (2.5 to 3.4)		23.4	27.3	3.9 (3.5 to 4.3)	
Ethnicity				0.14				0.006				0.085				0.003				0.97
Chinese	65.9	71.6	5.7 (5.4 to 6.1)		73.4	67.2	-6.2 (-6.6 to -5.7)		62.4	72.1	9.7 (9.3 to 10.1)		29.7	33.3	3.6 (3.1 to 4.0)		26.1	30.4	4.3 (3.9 to 4.7)	
Malay	57.7	64.8	7.1 (6.4 to 7.8)		69.1	63.0	-6.1 (-6.8 to -5.3)		54.0	65.9	11.9 (11.1 to 12.6)		23.0	27.1	4.1 (3.3 to 4.8)		12.6	15.2	2.6 (2.1 to 3.0)	
Indian	54.6	60.8	6.2 (5.4 to 7.1)		72.0	67.2	-4.8 (-5.7 to -3.9)		59.1	69.4	10.3 (9.5 to 11.2)		23.5	27.9	4.4 (3.5 to 5.2)		16.3	19.6	3.3 (2.7 to 3.8)	
Others	60.2	66.3	6.1 (4.9 to 7.2)		70.8	64.9	-5.9 (-7.2 to -4.6)		57.0	67.8	10.8 (9.5 to 12.1)		24.5	28.3	3.8 (2.6 to 5.1)		15.8	18.8	3.0 (2.3 to 3.8)	
Housing type				0.55				<0.001				<0.001				0.003				0.26
1~2 rooms HDB	60.3	66.9	6.6 (5.7 to 7.5)		70.3	66.1	-4.2 (-5.2 to -3.2)		57.7	70.1	12.4 (11.4 to 13.4)		24.9	29.8	4.9 (3.9 to 5.9)		26.2	30.2	4.0 (3.3 to 4.7)	
3~5 rooms HDB	63.2	69.2	6.0 (5.6 to 6.3)		72.4	66.3	-6.1 (-6.5 to -5.7)		61.0	71.1	10.1 (9.7 to 10.5)		28.0	31.7	3.7 (3.3 to 4.1)		22.5	26.3	3.8 (3.4 to 4.2)	
Condo or landed house	65.9	71.4	5.5 (4.8 to 6.2)		75.2	68.6	-6.6 (-7.4 to -5.8)		60.0	68.7	8.7 (7.9 to 9.5)		29.3	32.4	3.1 (2.3 to 3.9)		22.7	26.9	4.2 (3.7 to 4.8)	

Abbreviation: HbA_1c_, glycated haemoglobin; BP, blood pressure; LDL-C, low density lipoprotein cholesterol; 95% CI, 95% confidence interval; HDB, Housing and Development Board

^a^ Predictive margins were calculated using multivariate logistic generalized estimating equations (GEEs) regression for correlated outcomes, including categorical year of data collection and adjusting for age, gender, ethnicity, and housing type.

^b^ Participants younger than 45 years of age without complications (HbA_1c_, ≤6.5%) or with complications (≤7.0%); those 45 to 64 years of age without complications (HbA_1c_, ≤7.0%) or with complications (≤8.0%); and those 65 years of age or older without complications (HbA_1c_, ≤7.0%) or with complications(≤8.0%).

^c^ Defined by meeting individualized HbA_1c_, BP, and LDL

^d^ P value for the interaction between year of data collection and demographics

A smaller proportion of women achieved BP, LDL-C and combined targets relative to men in both 2013 and 2019. With the exception of BMI, the change in proportion of patients achieving risk factor control between 2013 to 2019 differed significantly by gender. Among the different ethnic groups, a greater proportion of Chinese patients achieved risk factor control in both 2013 and 2019. The Malay subgroup had the smallest proportion achieving BP, LDL-C and combined targets, and the Indian subgroup had the smallest proportion achieving individualized HbA1c targets. Temporal trends of CHD 10-year risk, and proportion of patients achieving BP and combined targets varied significantly by ethnicity.

Individuals living in lower-cost housing (1- to 2- room HDB flats) had poorer risk factor control except for BMI, and higher 10-year CHD risks than those living in higher-cost housing in 2013. But the disparity was smaller in terms of BP control, and was reversed in terms of LDL-C control in 2019. When HbA1c targets were defined as <7.0% (S3 Table), the direction of temporal trends in proportion achieving HbA1c and combined targets were similar across demographic subgroups.

After additionally accounting medication use and BMI in the models, demographic-related variation in the temporal trend of risk factor control persisted (S4 and S5 Tables).

### Changes in medication use

The adjusted proportion of medication use decreased for any glucose-lowering medication, metformin, and antihypertensives, and increased significantly for SGLT2 i, antiplatelet, insulin, and statins from 2013 to 2019, with the highest increase occurring for the SGLT2 i (12.4%,95%CI (12.2 to 12.6), P for linear trend <0.001) (S6 Table). Similar trends were shown for these medications among patients with uncontrolled risk factors (S7 Table).

## Discussion

In a series of cross-sectional surveys of the public sector diabetes registry data from 2013 and 2019 on more than 200,000 patients with diabetes in Singapore, we showed that despite some improvement in trends of glycemia and LDL-C levels over 6 years, gaps persisted in their control rates. Moreover, both BP levels and BP control worsened during this period. Additionally, 10-year CHD, stroke risk, and CVD mortality increased between 2013 and 2019. Temporal trends in the risk factor control and 10-year CHD and stroke risks varied by demographic subgroup. Compared with young adults, the subgroup of older adults had better glycemic control but poorer BP control and higher 10-year CHD and stroke risks in 2013. Temporal improvements in risk factor control were also smaller among the elderly, coupled with a greater increase in 10-year CHD and stroke risk over time.

Asian populations have a high risk of diabetes and related vascular disease [6]. In Asia, the number of people living with diabetes increased by 85% in the past 10 years [21, 22], and is projected to continue increasing by 25% from 250.2 million in 2019 to 311.6 million by 2045 [22]. Singapore ranked 2^th^ from the top worldwide in end-stage kidney disease attributable to diabetes in 2016 [23]. However, previous studies examining temporal trends of risk factor control among patients with type 2 diabetes have largely been performed in western populations [24–29].

The worsening of BP control over time is concerning, given the well-documented link between hypertension and diabetic complications [10]. The proportion of patients achieving BP targets decreased from 75.0% in 2013 to 67.7% in 2019, and the mean SBP increased by 2.4 mmHg during this period. Of note, trend analyses of population-wide data from the US also showed a concerning fall in BP control rates from 53.8% in 2013–2014 to 43.7% in 2017–2018 [30]. There are multiple barriers to controlled BP [31]. The observed reduction in antihypertensive medication use in the entire sample and those with uncontrolled BP during the same period might explain the increased SBP. However, adjustment for antihypertensive medication use over time did not significantly affect the trend. Neither did adjustment for BMI change over time. Other factors including medication nonadherence and unhealthy lifestyle such as high dietary sodium intake are also possible contributors to worsening trend in uncontrolled BP [31–33]. Because these data are not available, further studies are required to elucidate their contribution to this worsening trend among patients with diabetes.

Even a small increase in SBP of about 2 mm Hg may elevate the risk of CHD and stroke by 4 to 6% [34]. Thus, the increase in the 10-year CHD and stroke risk and all-cause and CVD mortality after adjusting for age and other demographics could be partly attributed to deterioration of BP control despite the improvement in glycaemic and LDL-C control over time. Moreover, compared with younger adults, older adults had a higher 10-year CHD and stroke risk in 2013, followed by a greater increase in the risk score over the subsequent 6 years. It might be explained by the age-related heterogeneity with regards to BP control because more pronounced deterioration of BP control was observed among older adults. Most alarming are the persistent, huge gaps in BP control rates in the entire cohort with only 67.7% controlled to the conventional target of <140/90 mmHg in 2019. Our findings call for immediate interventions for better hypertension management and cardiovascular risk reduction in patients with diabetes.

Consistent with previous studies [29, 35, 36], glycaemic control improved over time in the entire population, and remained so despite accounting for baseline socio-demographics, baseline and follow-up BMI, use of glucose lowering medications, and complications of diabetes, suggesting these factors do not explain the favourable trend. Singapore has launched a number of policy initiatives for a “War on Diabetes (WoD)” to reduce diabetes burden since 2016 [37]. Exposure to WoD initiatives has been reported to be associated with good health-seeking behaviour such as meeting dietary recommendations and participation in health screening [38], which might lead to the improved glycaemic control over time. Access to resources and medication adherence [39], though on which the impact of WoD has yet to be studied, could also play a part. Unfortunately, we were unable to assess these factors due to their unavailability. The reasons behind the improving trend deserve further studies.

We found that older adults had better glycaemic control, which is in line with findings from other studies [40–42]. Possible reasons could be that older patients may have better access to medical care, may be more motivated to receive care, are more adherent with medication use, and are easier to achieve individualized HbA1c target of ≤8% [41, 43] The greater gain at glycaemic control rate among the young adults is most likely due to their low initial control rate, leaving more room for improvement as compared with old adults.

In this study, a smaller proportion of women achieved BP, LDL-C, and combined targets compared to men. These findings are consistent with the less favorable CVD risk profiles in women reported previously among persons with diabetes in studies from the US and Italy [44, 45]. Our study found that ethnicity played a role in the degree of risk factor control. A smaller proportion of Malay patients achieved BP and LDL-C targets, and a smaller proportion of Indian patients achieved HbA1c targets compared to other ethnicities in both 2013 and 2019. In contrast, persistently better risk factor control was observed among Chinese vs. other ethnicities for all risk factors. Of note, these ethnic differences in risk factor control are not explained by BMI or medication use. It has been reported that Indian patients with diabetes had poorer medication adherence [46] and higher insulin resistance than other ethnic groups, likely causing poor glycaemic control [47]. The suboptimal risk factor control among Malay patients might be explained by their lower medication adherence [46] and unhealthy lifestyle factors that were not recorded in SDR [48]. However, the results highlight that greater efforts are needed to optimize risk factor control among women and non-Chinese ethnic groups.

Individuals from lower socioeconomic backgrounds as inferred by their housing type were less likely to achieve risk factor control, and had higher 10-year CHD risks compared to those from higher socioeconomic backgrounds in 2013. However, the social gradient in trends in risk factor control was smaller for BP control and reversed for LDL-C control in 2019, indicating greater improvement in risk-factor control among individuals with low income. Housing type is an indicator of socio-economic status [49] and is positively correlated with income in Singapore [50]. Individuals from higher socioeconomic backgrounds can afford a better quality of diabetes care and may have better medication adherence [51–53], leading to better risk factor profiles. Meanwhile, the more favorable change in risk factor control among individuals from lower socioeconomic backgrounds might be explained by the recent enhancement of certain healthcare schemes in Singapore. For example, the Community Health Assist Scheme (CHAS) was introduced in 2000 to provide low- and middle-income Singaporeans aged ≥ 65 years with better access to primary healthcare by bringing affordable services closer to them [54]. Still, large treatment gaps remain among individuals in lower socioeconomic strata in 2019, underscoring the necessity of more efforts to mitigate the impact of economic inequality on diabetes management.

The strengths of the study are the large sample of multi-ethnic diabetic patients of all socioeconomic strata from multiple institutions in Singapore, in-depth data capture with very little missing data on key variables, and excellent linkage with laboratory data. At the same time, the study has several limitations. First, smoking data were not comprehensively collected and the year of collection was unknown. Also, information on preventive care such as diabetes education, physical activity, and dietary pattern was not available. Therefore, we were unable to fully capture all aspects of temporal trends in risk factor control and preventive care. Second, UKPDS risk engines were not validated in our study population. The UKPDS risk engine has been shown to overestimate CHD and stroke risk among Chinese patients with type 2 diabetes [55, 56]. Also, we assumed that smoking status remained unchanged throughout the study period to calculate the UKPDS, likely leading to an inaccurate estimate of UKPDS risk. However, data form Singapore National Health Survey showed only a slight decline in prevalence of smoking from 2010 to 2019 [57]. We thus believe that the overestimation of the risk would not noticeably affect the direction of temporal trends of these indicators. Further study is needed with validated risk equations. Third, our data are not representative of all healthcare institutions of Singapore. However, SingHealth provides clinical services to approximately 50% of the Singapore population, hence the findings would be generalizable to the entire republic. Finally, some caution is warranted in generalizing our findings to adults with diabetes from other Asian countries due to the heterogeneity in clinical practice, health systems, and policy in different countries. However, we believe persistent gaps in risk factor control would be observed in many other high-income Asian countries and areas like Malaysia, Thailand, South Korea, Hong Kong, Taiwan, and especially in urban areas in mainland China with similar populations and healthcare infrastructure [58–63].

In conclusion, there was a worsening trend in BP control compared to an improving trend in the control of HbA1c and LDL-C from 2013 to 2019 in Singapore. Both 10-year CHD and stroke risk as well as CVD mortality showed a concerning increase over time, and risk factor control was suboptimal, indicating a wide gap between recommended and achieved targets in routine clinical practice. Young adults, women, non-Chinese ethnicity, and low-income individuals generally had worse risk factor control. Continued public health efforts are needed to address the treatment gaps among many individuals with diabetes and poorly controlled vascular risk in Singapore and most Asian countries.

## Supporting information

S1 Table Change in risk factor control further adjusted for medication use and body mass index among patients with diabetes. (DOCX)

S2 Table Adjusted change in proportions (95% confidence interval) for high coronary heart disease (CHD) risk, high stroke risk, and cardiovascular disease (CVD) mortality among patients with diabetes according to demographic characteristics. (DOCX)

S3 Table Adjusted change between 2013 and 2019 in proportions (95% confidence interval) achieving single or combined risk factor control among patients with diabetes according to demographic characteristics. (DOCX)

S4 Table Change further adjusted for medication use between 2013 and 2019 in proportions (95% confidence interval) achieving single or combined risk factor control among patients with diabetes according to demographic characteristics. (DOCX)

S5 Table Change further adjusted for medication use and body mass index between 2013 and 2019 in proportions a (95% confidence interval) achieving single or combined risk factor control among patients with diabetes according to demographic characteristics. (DOCX)

S6 Table Change in medication use among all patients with diabetes. (DOCX)

S7 Table Change in medication use among patients with diabetes who had uncontrolled risk factors. (DOCX)
