# Supplementary material for: Trends in cardiovascular risk factors and treatment goals in patients with diabetes in Singapore-analysis of the SingHealth Diabetes Registry
Source: PLoS One. 2021 Nov 8;16(11):e0259157. doi: 10.1371/journal.pone.0259157 (PMC8575178; doi:10.1371/journal.pone.0259157)
Supplement: S1 Table — (DOCX) [file pone.0259157.s001.docx]

S1 Table Change in risk factor control further adjusted for medication use and body mass index among patients with diabetes

| **Characteristics** | **2013  (n=** 86480 **)** | **2014  (n=**93132**)** | **2015 (n=**99404**)** | **2016 (n=**106144 **)** | **2017 (n=**110623**)** | **2018 (n=**127901**)** | **2019 (n=133180)** | **Adjusted Change from 2013 to 2019 based on model 1^a^ , % (95% CI)** | **Adjusted Change from 2013 to 2019 based on model 2^b^ , % (95% CI)** |
| --- | --- | --- | --- | --- | --- | --- | --- | --- | --- |
| **HbA1c** |  |  |  |  |  |  |  |  |  |
| <7.0%, n/N (%) | 37407/81535 (45.9) | 41239/84971 (48.5) | 41977/90129 (46.6) | 42906/95026 (45.2) | 37424/95239 (39.3) | 59649/115123 (51.8) | 62237/120947 (51.5) | 3.6 (3.2 to 3.4) | 4.9 (4.4, 5.5) |
| Missing, n | 4945 | 8161 | 9275 | 11118 | 15384 | 12778 | 12233 |  |  |
| **Blood pressure (mmHg)** |  |  |  |  |  |  |  |  |  |
| SBP/DBP<140/90, n/N (%) | 58092/77465 (75.0) | 63502/87119 (72.9) | 66350/92378 (71.8) | 72988/101166 (72.1) | 76536/ 109027 (70.2) | 86562/126254 (68.6) | 88653/130968 (67.7) | -6.5 (-6.8 to -6.1) | -3.6 (-4.1,-3.1) |
| Missing, n | 9015 | 6013 | 7026 | 4978 | 1596 | 1647 | 2212 |  |  |
| **LDL cholesterol (**mg/dl) |  |  |  |  |  |  |  |  |  |
| <100, n/N (%) | 48008/74074  (64.8) | 47918/ 76064 (63.0) | 52562/ 81748 (64.3) | 59039/ 86870 (68.0) | 66566/ 88821 (74.9) | 75516/ 103908 (72.7) | 78056/108072 (72.2) | 10.0 (9.6 to 10.4) | 9.9 (9.4, 10.5) |
| Missing, n | 12406 | 17068 | 17656 | 19274 | 21802 | 23993 | 25108 |  |  |
| **HbA1c, blood pressure, and LDL cholesterol** |  |  |  |  |  |  |  |  |  |
| Targets achieved ^c^ n/N (%) | 17733/79231 (22.4) | 18793/ 84035 (22.4) | 19115/89880 (21.3) | 20521/ 96075 (21.4) | 19565/99611 (19.6) | 29625/ 116328 (25.5) | 29534/121152 (24.4) | 2.0 (1.6 to 2.3) | 3.0 (2.5, 3.5) |
| Missing ^C^, n | 7249 | 9097 | 9524 | 10069 | 11012 | 11573 | 12028 |  |  |

N was the total number of patients with the corresponding measurement
Abbreviation: 95% CI, 95% confidence interval; SBP, systolic blood pressure; DBP, diastolic blood pressure; LDL, low density lipoprotein.
a Model 1,adjusted for age, gender, ethnicity, housing type, and medication use (glucose-lowering medication for HbA1c<7.0%, antihypertensive use for SBP/DBP<140/90, lipid-lowering medication use for LDL<100, use of all three medication for achieving combined targets of HbA1c, blood pressure, and LDL cholesterol)
b Model2, adjusted for variables in model 1+ body mass index
^C^ HbA1c<7.0%, SBP≤140&DBP≤90 mmHg, and LDL<100mg/dl
