# Supplementary material for: Trends in cardiovascular risk factors and treatment goals in patients with diabetes in Singapore-analysis of the SingHealth Diabetes Registry
Source: PLoS One. 2021 Nov 8;16(11):e0259157. doi: 10.1371/journal.pone.0259157 (PMC8575178; doi:10.1371/journal.pone.0259157)
Supplement: S2 Table — (DOCX) [file pone.0259157.s002.docx]

S2 Table Adjusted change in proportions ^a^ (95% confidence interval) for high coronary heart disease (CHD), high stroke risk, and cardiovascular disease (CVD) mortality among patients with diabetes according to demographic characteristics

| Characteristics |  |  | CHD UKPDS risk ≥15% (n=65019) |  |  |  |  | Stroke UKPDS risk ≥15% (n=71594) |  |  |  |  | CVD mortality rate (n=182638) |  |
| --- | --- | --- | --- | --- | --- | --- | --- | --- | --- | --- | --- | --- | --- | --- |
|  | Year 2013, % | Year 2019, % | Absolute change from 2013 to 2019, % (95% CI) | P for  interaction ^b^ |  | Year 2013, % | Year 2019, % | Absolute change from 2013 to 2019, % (95% CI) | P for  interaction ^b^ |  | Year 2013, % | Year 2019, % | Absolute change from 2013 to 2019, % (95% CI) | P for  interaction ^b^ |
| Age (yr) |  |  |  | <0.001 |  |  |  |  | <0.001 |  |  |  |  | 0.91 |
| 18~44 | 12.3 | 11.0 | -1.3 (-2.4 to -0.2) |  |  | 2.5 | 4.3 | 1.8 (0.9 to 2.5) |  |  | 0.2 | 0.4 | 0.2 (-0.0, 0.4) |  |
| 45~64 | 27.5 | 29.5 | 2.0 (1.4 to 2.6) |  |  | 7.6 | 18.7 | 11.1 (10.7 to 11.6) |  |  | 0.4 | 0.8 | 0.3 (0.2, 0.4) |  |
| 65 and over | 49.3 | 57.3 | 8.0 (7.3 to 8.6) |  |  | 24.5 | 54.3 | 29.8 (29.4 to 30.3) |  |  | 1.3 | 2.4 | 1.1 (0.8, 1.3) |  |
| Gender |  |  |  | <0.001 |  |  |  |  | 0.57 |  |  |  |  | 0.70 |
| Male | 56.6 | 61.4 | 4.8 (4.2 to 5.5) |  |  | 18.3 | 41.0 | 22.7 (22.2 to 23.1) |  |  | 1.0 | 1.8 | 0.8 (0.6, 1.0) |  |
| Female | 19.8 | 24.8 | 5.0 (4.4 to 5.5) |  |  | 12.5 | 32.6 | 20.1 (19.6 to 20.5) |  |  | 0.8 | 1.4 | 0.6 (0.5, 0.8) |  |
| Ethnicity |  |  |  | <0.001 |  |  |  |  |  |  |  |  |  | 0.015 |
| Chinese | 35.3 | 40.8 | 5.5 (4.9 to 6.0) |  |  | 16.0 | 38.0 | 22.0 (21.6 to 22.4) | 0.18 |  | 0.8 | 1.5 | 0.7 (0.6, 0.8) |  |
| Malay | 41.1 | 44.3 | 3.2 (2.3 to 4.2) |  |  | 12.5 | 32.5 | 20.0 (19.2 to 20.7) |  |  | 1.3 | 1.9 | 0.6 (0.3, 0.9) |  |
| Indian | 40.6 | 45.0 | 4.4 (3.3 to 5.6) |  |  | 13.7 | 33.8 | 20.1 (19.2 to 20.9) |  |  | 1.0 | 1.8 | 0.8 (0.4, 1.1) |  |
| Others | 41.8 | 45.3 | 3.5 (1.8 to 5.3) |  |  | 15.7 | 36.8 | 21.1 (19.9 to 22.4) |  |  | 1.0 | 2.2 | 1.2 (0.7, 1.7) |  |
| Housing type |  |  |  | 0.005 |  |  |  |  | 0.85 |  |  |  |  | 0.56 |
| 1~2 rooms HDB | 43.7 | 46.9 | 3.2 (1.9 to 4.5) |  |  | 19.1 | 41.6 | 22.5 (21.6 to 23.5) |  |  | 1.3 | 2.2 | 0.9 (0.5, 1.3) |  |
| 3~5 rooms HDB | 36.3 | 41.4 | 5.1 (4.6 to 5.6) |  |  | 14.9 | 36.2 | 21.3 (20.9 to 21.7) |  |  | 0.9 | 1.6 | 0.7 (0.6, 0.9) |  |
| Condo or landed house | 35.8 | 41.3 | 5.5 (4.5 to 6.6) |  |  | 15.5 | 37.0 | 21.5 (20.7 to 22.2) |  |  | 0.7 | 1.2 | 0.5 (0.2, 0.7) |  |

Abbreviation: UKPDS, United Kingdom Prospective Diabetes Study, 95% CI, 95% confidence interval; HDB, Housing and Development Board

a Predictive margins were calculated using multivariate logistic generalized estimating equations (GEEs) regression for correlated outcomes, including categorical year of data collection and adjusting for age, sex, ethnicity, and housing type

b P value for the interaction between year of data collection and demographics
