# Supplementary material for: Trends in cardiovascular risk factors and treatment goals in patients with diabetes in Singapore-analysis of the SingHealth Diabetes Registry
Source: PLoS One. 2021 Nov 8;16(11):e0259157. doi: 10.1371/journal.pone.0259157 (PMC8575178; doi:10.1371/journal.pone.0259157)
Supplement: S3 Table — (DOCX) [file pone.0259157.s003.docx]

Table S3 Adjusted change between 2013 and 2019 in proportions ^a^ (95% confidence interval) achieving single or combined risk factor control among patients with diabetes according to demographic characteristics

| Characteristics |  |  | Glycated haemoglobin<7.0% (n=160178) |  |  | Controlled all 3 risk factors ^b^ (n=165380) | | | |
| --- | --- | --- | --- | --- | --- | --- | --- | --- | --- |
|  | Year 2013, % | Year 2019, % | Absolute change from 2013 to 2019, % (95% CI) | P for  interaction ^d^ |  | Year 2013, % | Year 2019, % | Absolute change from 2013 to 2019, % (95% CI) | P for interaction ^d^ |
| Age (yr) |  |  |  | <0.001 |  |  |  |  | <0.001 |
| 18~44 | 37.6 | 42.3 | 4.7 (3.5, 6.0) |  |  | 10.5 | 13.0 | 2.5 (1.6, 3.4) |  |
| 45~64 | 43.2 | 47.6 | 4.4 (3.9, 4.9) |  |  | 18.1 | 22.0 | 3.8 (3.4, 4.3) |  |
| 65 and over | 53.6 | 56.4 | 2.7 (2.3, 3.2) |  |  | 24.3 | 25.3 | 1.0 (0.6,1.4) |  |
| Gender |  |  |  | 0.87 |  |  |  |  | 0.016 |
| Male | 47.9 | 51.4 | 3.5 (3.1, 4.0) |  |  | 22.1 | 24.6 | 2.5 (2.1, 3.0) |  |
| Female | 49.4 | 52.9 | 3.5 (3.0,3.9) |  |  | 20.4 | 22.2 | 1.8 (1.4, 2.3) |  |
| Ethnicity |  |  |  | <0.001 |  |  |  |  | 0.094 |
| Chinese | 51.3 | 54.8 | 3.5 (3.1, 3.9) |  |  | 22.8 | 25.0 | 2.2 (1.8, 2.6) |  |
| Malay | 43.2 | 47.7 | 4.5 (3.8, 5.2) |  |  | 17.1 | 19.6 | 2.5 (1.9, 3.2) |  |
| Indian | 39.7 | 42.1 | 2.3 (1.5, 3.2) |  |  | 16.8 | 18.8 | 1.9 (1.3, 2.7) |  |
| Others | 45.1 | 48.3 | 3.2 (2.0, 4.4) |  |  | 18.0 | 20.2 | 2.2 (1.1, 3.3) |  |
| Housing type |  |  |  | 0.009 |  |  |  |  | 0.003 |
| 1~2 rooms HDB | 46.1 | 49.8 | 3.7 (2.7, 4.7) |  |  | 18.8 | 21.5 | 2.7 (1.9, 3.6) |  |
| 3~5 rooms HDB | 48.5 | 52.1 | 3.6 (3.2, 4.0) |  |  | 21.3 | 23.5 | 2.3 (1.9, 2.7) |  |
| Condo or landed house | 52.0 | 54.4 | 2.4 (1.6, 3.2) |  |  | 22.6 | 23.9 | 1.3 (0.6, 2.0) |  |
|  |  |  |  |  |  |  |  |  |  |

Abbreviation: 95% CI, 95% confidence interval; HDB, Housing and Development Board

^a^ Predictive margins were calculated using multivariate logistic generalized estimating equations (GEEs) regression for correlated outcomes, including categorical year of data collection and adjusting for age, gender, ethnicity, and housing type.

^b^ Defined by meeting HbA_1c_<7.0%, systolic blood pressure (SBP)/diastolic blood pressure (DBP) <140/90 mmHg, and low-density lipoprotein cholesterol (LDL-C) <100 mg/dl

^d^ P value for the interaction between year of data collection and demographics
