# Supplementary material for: Trends in cardiovascular risk factors and treatment goals in patients with diabetes in Singapore-analysis of the SingHealth Diabetes Registry
Source: PLoS One. 2021 Nov 8;16(11):e0259157. doi: 10.1371/journal.pone.0259157 (PMC8575178; doi:10.1371/journal.pone.0259157)
Supplement: S4 Table — (DOCX) [file pone.0259157.s004.docx]

S4 Table Change **further adjusted for medication use** between 2013 and 2019 in proportions ^a^ (95% confidence interval) achieving single or combined risk factor control among patients with diabetes according to demographic characteristics

| Characteristics |  |  | Met individualized HbA_1c_ target ^b^ (n=160178) |  |  |  | BP<140/90 mmHg  (n=176809) |  |  |  | LDL-C<100mg/dL  (n=150692) |  | Controlled all 3 risk factors^c^ (n=164305) | | | |
| --- | --- | --- | --- | --- | --- | --- | --- | --- | --- | --- | --- | --- | --- | --- | --- | --- |
|  | Year 2013, % | Year 2019, % | Absolute change from 2013 to 2019, % (95% CI) | P for  interaction ^d^ | Year 2013,% | Year 2019, % | Absolute change from 2013 to 2019, % (95% CI) | P for interaction ^d^ | Year 2013, % | Year 2019, % | Absolute change from 2013 to 2019, % (95% CI) | P for interaction ^d^ | Year 2013, % | Year 2019, % | Absolute change from 2013 to 2019, % (95% CI) | P for interaction ^d^ |
| Age (yr) |  |  |  | <0.001 |  |  |  | <0.001 |  |  |  | <0.001 |  |  |  | <0.001 |
| 18~44 | 28.6 | 36.0 | 7.4 (6.2 to 8.7) |  | 78.6 | 74.3 | -4.3 (-5.5 to -3.1) |  | 42.4 | 54.3 | 11.9 (10.4 to 13.4) |  | 9.0 | 12.3 | 3.3 (2.3 to 4.3) |  |
| 45~64 | 56.0 | 63.5 | 7.5 (7.0 to 8.0) |  | 75.0 | 69.9 | -5.1 (-5.6 to -4.6) |  | 55.8 | 66.8 | 11.0 (10.5 to 11.5) |  | 25.2 | 31.0 | 5.8 (5.3 to 6.3) |  |
| 65 and over | 71.1 | 75.8 | 4.7 (4.4 to 5.1) |  | 70.8 | 62.8 | -8.0 (-8.5 to -7.6) |  | 67.6 | 75.8 | 8.2 (7.8 to 8.6) |  | 32.2 | 34.2 | 2.0 (1.5 to 2.4) |  |
| Gender |  |  |  | 0.03 |  |  |  | <0.001 |  |  |  | 0.003 |  |  |  | <0.001 |
| Male | 63.7 | 69.8 | 6.1 (5.7 to 6.6) |  | 74.0 | 67.9 | -6.1 (-6.6 to -5.6) |  | 63.2 | 72.8 | 9.6 (9.1 to 10.0) |  | 30.3 | 34.5 | 4.2 (3.7 to 4.7) |  |
| Female | 62.0 | 67.8 | 5.8 (5.3 to 6.2) |  | 71.5 | 64.1 | -7.4 (-7.9 to -6.9) |  | 60.2 | 69.5 | 9.3 (8.8 to 9.7) |  | 26.7 | 29.5 | 2.8 (2.3 to 3.3) |  |
| Ethnicity |  |  |  | 0.25 |  |  |  | 0.006 |  |  |  | 0.13 |  |  |  | 0.006 |
| Chinese | 64.9 | 70.7 | 5.8 (5.4 to 6.2) |  | 73.6 | 66.7 | -6.9 (-7.3 to -6.5) |  | 63.6 | 72.6 | 9.0 (8.7 to 9.4) |  | 30.3 | 33.7 | 3.4 (2.9 to 3.8) |  |
| Malay | 58.8 | 65.6 | 6.8 (6.1 to 7.5) |  | 69.6 | 62.8 | -6.8 (-7.5 to -6.0) |  | 54.4 | 65.6 | 11.2 (10.4 to 11.9) |  | 23.2 | 27.0 | 3.8 (3.1 to 3.5) |  |
| Indian | 56.0 | 62.0 | 6.0 (5.2 to 6.8) |  | 71.8 | 66.2 | -5.6 (-6.4 to -4.7) |  | 60.3 | 69.9 | 9.6 (8.7 to 10.4) |  | 24.5 | 28.7 | 4.2 (3.4 to 5.0) |  |
| Others | 61.0 | 66.9 | 5.9 (4.7 to 7.0) |  | 71.1 | 64.4 | -6.7 (-8.0 to -5.4) |  | 57.9 | 68.1 | 10.2 (8.9 to 11.5) |  | 25.0 | 28.7 | 3.7 (2.5 to 5.0) |  |
| Housing type |  |  |  | 0.91 |  |  |  | <0.001 |  |  |  | <0.001 |  |  |  | 0.004 |
| 1~2 rooms HDB | 60.4 | 66.7 | 6.3 (5.4 to 7.2) |  | 70.6 | 65.7 | -4.9 (-5.9 to -3.9) |  | 58.5 | 70.2 | 11.7 (10.8 to 12.7) |  | 25.2 | 29.8 | 4.6 (3.7 to 5.6) |  |
| 3~5 rooms HDB | 62.9 | 68.8 | 5.9 (5.6 to 6.3) |  | 72.7 | 65.9 | -6.8 (-7.3 to -6.4) |  | 62.0 | 71.5 | 9.5 (9.1 to 9.8) |  | 28.5 | 32.0 | 3.5 (3.1 to 3.9) |  |
| Condo or landed house | 64.6 | 70.3 | 5.7 (5.0 to 6.5) |  | 75.0 | 67.6 | -7.4 (-8.2 to -6.6) |  | 61.8 | 69.7 | 7.9 (7.1 to 8.7) |  | 30.4 | 33.4 | 3.0 (2.2 to 3.8) |  |

Abbreviation: HbA_1c_, glycated haemoglobin; BP, blood pressure; LDL-C, low density lipoprotein cholesterol; 95% CI, 95% confidence interval; HDB, Housing and Development Board

^a^ Predictive margins were calculated using multivariate logistic generalized estimating equations (GEEs) regression for correlated outcomes, including categorical year of data collection and adjusting for age, gender, ethnicity, housing type, antidiabetic medication use (for the outcomes of individualized HbA_1c_ target and controlled all 3 risk factors), antihypertensive use (for the outcomes of BP<140/90 mmHg and controlled all 3 risk factors), and lipid-lowering medication (for the outcomes of LDL-C<100mg/dL and controlled all 3 risk factors)

^b^ Participants younger than 45 years of age without complications (HbA_1c_, ≤6.5%) or with complications (≤7.0%); those 45 to 64 years of age without complications (HbA_1c_, ≤7.0%) or with complications (≤8.0%); and those 65 years of age or older without complications (HbA_1c_, ≤7.0% ~~)~~ or with complications(≤8.0%).

^C^ Defined by meeting individualized HbA_1c_, BP, and LDL-C

^d^ P value for the interaction between year of data collection and demographics
