# Supplementary material for: Trends in cardiovascular risk factors and treatment goals in patients with diabetes in Singapore-analysis of the SingHealth Diabetes Registry
Source: PLoS One. 2021 Nov 8;16(11):e0259157. doi: 10.1371/journal.pone.0259157 (PMC8575178; doi:10.1371/journal.pone.0259157)
Supplement: S6 Table — (DOCX) [file pone.0259157.s006.docx]

S6 Table Change in medication use among all patients with diabetes

|  | **2013  (n=** 86480**)** | **2014  (n=**93132**)** | **2015 (n=**99404**)** | **2016 (n=**106144**)** | **2017 (n=**110623**)** | **2018 (n=**127901**)** | **2019 (n=133180)** | **Unadjusted change from 2013 to 2019, % (95% CI)^a^** | **Adjusted change from 2013 to 2019, % (95% CI)^b^** | **P for linear trend^c^** |
| --- | --- | --- | --- | --- | --- | --- | --- | --- | --- | --- |
| Use of antidiabetic medication, n (%) | 69345 (80.2) | 72949 (78.3) | 76180 (76.6) | 80606(75.9) | 82584 (74.7) | 94053 (73.5) | 99613 (74.8) | -1.7 (-2.0 to -1.4) | -1.2 (-1.6, -0.9) | <0.001 |
| Use of metformin, n (%) | 58990 (68.2) | 62062 (66.6) | 65350 (65.7) | 69507 (65.5) | 71634 (64.8) | 79465 (62.1) | 84425 (63.4) | -4.2 (-4.5 to -3.9) | -3.2 (-3.6, -2.9) | <0.001 |
| Use of SGLT2 inhibitor, n (%) | 0 | 294 (0.3) | 780 (0.8) | 1652 (1.6) | 7062 (6.4) | 13070 (10.2) | 18282 (13.7) | 12.4 (12.2 to 12.6) ^d^ | 12.4 (12,3, 12.6)^d^ | <0.001 |
| Use of insulin, n (%) | 8974 (10.4) | 9907 (10.6) | 10552 (10.6) | 11039 (10.4) | 10332 (9.3) | 13122 (10.3) | 15723 (11.8) | 3.6 (3.4 to 3.9) | 3.7 (3.4, 3.9) | <0.001 |
| Use of statin, n (%) | 68181(78.8) | 75727 (81.3) | 80776 (81.3) | 86182 (81.2) | 88854 (80.3) | 100733 (78.8) | 105523 (79.2) | 2.9 (2.6 to 3.2) | 2.3 (2.0, 2.6) | <0.001 |
| Use of any antihypertensives, n (%) | 73735 (85.3) | 77800 (83.5) | 81582 (82.1) | 85431 (80.5) | 88547 (80.0) | 97861 (76.5) | 102817 (77.2) | -4.5 (-4.8 to -4.2) | -6.4 (-6.7, -6.1) | <0.001 |
| Use of antiplatelet, n (%) | 25875 (29.9) | 27367 (29.4) | 28773 (28.9) | 32016 (30.2) | 33450 (30.2) | 37769 (29.5) | 40389 (30.3) | 5.4 (5.2, 5.7) | 4.0 (3.7, 4.3) | <0.001 |

Abbreviation: 95% CI, 95% confidence interval, SGLT2, Sodium-glucose co-transporter-2
^a^ Predictive margins were calculated from univariate logistic generalized estimating equations (GEEs) regression for correlated outcomes with categorical year of data collection as the independent variable
^b^ Predictive margins were calculated using multivariate logistic generalized estimating equations (GEEs) regression for correlated outcomes, including categorical year of data collection in the model and adjusting for age, gender, ethnicity, and housing type.
^c^ P value for linear trend was calculated using multivariate logistic generalized estimating equations (GEEs) regression for correlated outcomes including continuous year of data collection in the model and adjusting for age, gender, ethnicity, and housing type.
^d^ change from 2014 to 2019 because no patients used SGLT2 inhibitor in 2013.
