# Supplementary material for: Trends in cardiovascular risk factors and treatment goals in patients with diabetes in Singapore-analysis of the SingHealth Diabetes Registry
Source: PLoS One. 2021 Nov 8;16(11):e0259157. doi: 10.1371/journal.pone.0259157 (PMC8575178; doi:10.1371/journal.pone.0259157)
Supplement: S7 Table — (DOCX) [file pone.0259157.s007.docx]

S7 Table Change in medication use among patients with diabetes who had uncontrolled risk factors

|  | | **2013** | | **2014** | | **2015** | | **2016** | | **2017** | | **2018** | | **2019** | | **Unadjusted change from 2013 to 2019, % (95% CI)^a^** | | **Adjusted change from 2013 to 2019, % (95% CI)^b^** | **P for linear trend^c^** |  |
| --- | --- | --- | --- | --- | --- | --- | --- | --- | --- | --- | --- | --- | --- | --- | --- | --- | --- | --- | --- | --- |
| **Among patients with uncontrolled HbA1c (≥7.0%)** | | | | | | | |  | |  | |  | |  | |  | |  | |  |
| Use of antidiabetic medication, n/N (%) | | 41999/44128  (95.2) | | 41573/43732  (95.1) | | 45620/48152  (94.7) | | 49310/52120  (94.6) | | 54622/57815  (94.5) | | 51489/55474  (92.8) | | 54830/58710  (93.4) | | -0.8 (-1.1 to -0.5) | | -0.7 (-1.0, -0.3) | | <0.001 |
| Use of metformin, n/N (%) | | 36846 /44128  (83.5) | | 36236/43732   (82.9) | | 40170/48152  (83.4) | | 43661 /52120  (83.8) | | 48732/57815  (84.3) | | 43732/55474  (78.9) | | 46764/58710 (79.7) | | -3.4 (-3.8 to -2.9) | | -2.5 (-2.9, -2.0) | | <0.001 |
| Use of SGLT2 inhibitor, n/N (%) | | 0 | | 226/43732  (0.5) | | 581/48152  (1.2) | | 1235/52120  (2.4) | | 6222/57815  (10.8) | | 10562/55474  (19.0) | | 14115/58710  (24.1) | | 22.4 (22.1 to 22.7) ^d^ | | 22.7 (22.4, 23.0.)^d^ | | <0.001 |
| Use of insulin, n/N (%) | | 7108/44128  (16.1) | | 7598/43732 (17.4) | | 8028/48152  (16.7) | | 8332/52120  (16.0) | | 7693/57815  (13.3) | | 9274/55474  (16.7) | | 10766/58710 (18.3) | | 7.2 (6.8 to 7.5) | | 7.1 (6.8, 7.4) | | <0.001 |
| **Among patients with uncontrolled LDL-C (≥100 mg/dl)** | | | | | |  | |  | |  | |  | |  | |  | |  | |  |
| Use of statin, n/N (%) | | 19448/26006 (74.6) | | 22501/28146 (79.9) | | 23319/29186  (79.9) | | 22106/27831 (79.4) | | 17342/22255 (77.9) | | 20900/28392 (73.6) | | 21885/30016  (72.9) | | 4.7 (4.2 to 5.2) | | 4.1 (3.6, 4.6) | | <0.001 |
| **Among patients with uncontrolled BP (SBP/DPB≥140/90 mmHg)** | | | | | | | | | | | |  | |  | |  | |  | |  |
| Use of any antihypertensives, n/N (%) | | 18154/19373 (93.7) | | 21615/23617 (91.5) | | 23381/26028 (89.8) | | 24854/28178 (88.2) | | 28174/32491 (86.7) | | 34114/39692 (85.9) | | 36697/42315 (86.7) | | -8.9 (-9.8 to -8.1) | | -9.3 (-10.1, -8.4) | | <0.001 |

N was the total number of patients with the corresponding uncontrolled risk factors
Abbreviation: 95% CI, 95% confidence interval, SGLT2, Sodium-glucose co-transporter-2, LDL-C, low-density lipoprotein cholesterol, BP, blood pressure, SBP, systolic BP, DBP, diastolic BP.
^a^ Predictive margins were calculated from univariate logistic generalized estimating equations (GEEs) regression for correlated outcomes with categorical year of data collection as the independent variable
^b^ Predictive margins were calculated using multivariate logistic generalized estimating equations (GEEs) regression for correlated outcomes, including categorical year of data collection in the model and adjusting for age, gender, ethnicity, and housing type.
^c^ P value for linear trend was calculated using multivariate logistic generalized estimating equations (GEEs) regression for correlated outcomes including continuous year of data collection in the model and adjusting for age, gender, ethnicity, and housing type.
^d^ change from 2014 to 2019 because no patients used SGLT2 inhibitor in 2013.
